# Upgrading Model Selection Criteria with Goodness of Fit Tests for Practical Applications

**DOI:** 10.3390/e22040447

**Published:** 2020-04-15

**Authors:** Riccardo Rossi, Andrea Murari, Pasquale Gaudio, Michela Gelfusa

**Affiliations:** 1 Department of Industrial Engineering, University of Rome “Tor Vergata”, via del Politecnico 1, 01100 Roma, Italy; gaudio@ing.uniroma2.it (P.G.); gelfusa@ing.uniroma2.it (M.G.); 2 Consorzio RFX (CNR, ENEA, INFN, Università di Padova, Acciaierie Venete SpA), Corso Stati Uniti 4, 35127 Padova, Italy; Andrea.murari@euro-fusion.org

**Keywords:** model selection criteria, Bayesian information criterion (BIC), Akaike information criterion (AIC), Shannon entropy, goodness of fit tests, Kolmogorov–Smirnov test

## Abstract

The Bayesian information criterion (BIC), the Akaike information criterion (AIC), and some other indicators derived from them are widely used for model selection. In their original form, they contain the likelihood of the data given the models. Unfortunately, in many applications, it is practically impossible to calculate the likelihood, and, therefore, the criteria have been reformulated in terms of descriptive statistics of the residual distribution: the variance and the mean-squared error of the residuals. These alternative versions are strictly valid only in the presence of additive noise of Gaussian distribution, not a completely satisfactory assumption in many applications in science and engineering. Moreover, the variance and the mean-squared error are quite crude statistics of the residual distributions. More sophisticated statistical indicators, capable of better quantifying how close the residual distribution is to the noise, can be profitably used. In particular, specific goodness of fit tests have been included in the expressions of the traditional criteria and have proved to be very effective in improving their discriminating capability. These improved performances have been demonstrated with a systematic series of simulations using synthetic data for various classes of functions and different noise statistics.

## 1. Introduction to Model Selection Criteria Based On Bayes and Information Theory

The selection of the most appropriate model, to describe the phenomena under study, is a major concern of modern science [1,2]. Statisticians have naturally been involved in this task, and so it is not surprising that many statistical approaches have been proposed over the years for dealing with this key issue. Indeed, model selection has been investigated extensively from both frequentist and Bayesian perspectives. Many tools for identifying the “best model” among a set of candidates have been suggested in the literature [3,4]. Two of the most widely used model selection families of indicators are the Akaike information criterion (AIC) [5] and the Bayesian information criterion (BIC) [6]. The AIC is an information-theoretic indicator based on the Kullback–Leibler Divergence [7]; it essentially quantifies the information lost by a given model. Therefore, the basic principle underlying the AIC criterion is the assumption that the less information a model loses, the higher is its quality. Bayesian theory informs the BIC criterion, which is designed to maximize the posterior probability of a model given the data [7].

The theoretical derivations of these metrics result in the following unbiased forms of the criteria:

(1) AIC=−2ln(L)+2k

(2) BIC=−2ln(L)+kln(n)

where *L* is the likelihood of the model given the data, *k* the number of estimated parameters in the model, and *n* the number of entries in the database. Both AIC and BIC metrics are basically cost functions, which have to be minimized; they favor models with a high likelihood but implement a penalty for complexity (the term proportional to *k*).

The AIC and BIC criteria are very well known and have found many interesting applications. On the other hand, their original formulation is not necessarily easy to implement in practice. A very delicate part is the likelihood of the models, which can be virtually impossible to calculate. This difficulty can be due to various causes, ranging from the type of noise affecting the data to the nature of the models to be tested and the “a priori “information about their properties [8]. To overcome the practical difficulties of calculating the likelihood, one typically makes recourse to the traditional assumption that the model and data errors are identically distributed and independently sampled from a normal distribution. If this hypothesis is valid, it can be demonstrated that the AIC can be written (up to an additive constant, which depends only on the number of entries in the database and not on the model):

(3) AIC=n⋅ln(MSE)+2k

where $\sigma_{\left(\epsilon \right)}{}^{2}$ is the variance of the residuals.

Similar assumptions allow expressing the BIC criterion as:

(4) $$BIC=n\cdot \ln\left(\sigma_{\left(\epsilon \right)}{}^{2}\right)+k\cdot \ln\left(n\right)$$

where MSE is the mean-squared error of the residuals.

In Equations (3) and (4), derived, for example, in [7], the MSE and variance are calculated based on the residuals and the differences between the data and the estimates of the models.

As can be easily appreciated by inspection of Equations (3) and (4), which constitute the most widely used forms of AIC and BIC, the statistical information, originally provided by the likelihood, is reduced to the MSE and variance of the residuals. The natural question is, therefore, whether some additional statistical information about the distribution of the residuals could be taken into account and improve the performance of the two criteria. The practical importance of this question is not to be underestimated because, in many applications, the assumptions leading to expressions (3) and (4) are clearly violated. The first attempt in this direction, reported in [9], relied on the Shannon entropy as an indicator of the residual distribution. The encouraging results, obtained with this upgrade, has motivated the insertion of more sophisticated summary statistics of the distribution of the residuals into the AIC and BIC, to see whether they could further increase their performance. The paper described this line of investigation in detail and is structured as follows. The goodness of fit tests, implemented to improve AIC and BIC, are described in Section 2. The families of models and the typologies of statistical noise investigated are the subjects of Section 3. The following Section 4 describes the results obtained for three very important families of models: exponential functions, polynomials, and power laws. Section 5 reports the results for a scan of the noise amplitude. Summary and future developments are the subjects of the last Section 6.

## 2. Model Selection and Goodness of Fit Tests

As mentioned in the introduction, a previous attempt to improve the practical implementation of AIC and BIC was based on the Shannon entropy of the residuals. The main idea behind that approach was the observation that, if a model was perfect, the residuals should reflect the statistics of the noise affecting the data. Assuming additive random noise and other things being equal, models, whose residuals present a more uniform probability density function (pdf), should, therefore, be preferred. Indeed, the residuals of inferior models should contain patterns present in the data and not properly identified by the models. The Shannon entropy *H* can be interpreted as an indicator of how uniform is the distribution of the residuals and can, therefore, be included in the AIC and BIC to favor models with higher values of *H*. In this perspective, the following versions of the BIC and AIC criteria were tested:

(5) $$BIC_{H}=n\cdot \ln\left(\frac{\sigma_{\left(\epsilon \right)}{}^{2}}{H}\right)+k\cdot \ln\left(n\right)$$

(6) $$AIC_{H}=n\cdot \ln\left(\frac{MSE}{H}\right)+2k$$

where *H* = −$\sum_{i}p_{i}\;lnp_{i}$ indicates the Shannon entropy of the residuals, and *p_i_* is the probability of the i-th residual.

These new versions of the criteria clearly outperformed the traditional version of the AIC and BIC, and they had also asymptotic convergence [9]. On the other hand, entropy is a quite crude indicator of the residual distributions. Moreover, in many practical applications, the statistics of the noise is not necessarily Gaussian. It was, therefore, reasonable to investigate whether more sophisticated tests could further improve the discriminatory power of the AIC and BIC criteria. To this end, various goodness of fit tests had been implemented. The main rationale was that, very often, it was possible to determine experimentally the actual statistics of the noise affecting the data. At this point, better models should present residuals with a distribution more similar to the one of the noise.

The goodness of fit tests investigated in this work were:

1. Chi-squared;
2. Anderson Darling;
3. Kolmogorov–Smirnov.

The null distribution of all these statistics is calculated under the null hypothesis that the sample is drawn from the reference distribution, the pdf of the noise in our case.

The “chi-squared test”, or *χ*^2^ test, implemented in this paper was the Pearson’s chi-squared test [10], which is often used to determine whether there is a statistically significant difference between two probability distribution functions (pdfs). In our application, as already discussed, the two distributions to be compared were one of the residuals and one of the noises affecting the data.

The “Anderson–Darling” test can also be used to assess whether a sample is drawn from a specified distribution function [11]. It is based on the fact that, assuming the data does arise from this distribution, its cumulative distribution function (CDF) is expected to follow the one, which can be derived by the original distribution. In our application, the residuals could be then tested for uniformity with a distance test [12]. The test statistics could then be compared with the critical values of the distribution expected on the basis of the knowledge of the noise.

The “Kolmogorov–Smirnov” test (K–S test) is another nonparametric test to determine the equality between one-dimensional probability distributions or, as in our case, between a pdf and a sample [13]. The statistic of the K–S quantifies a distance between the empirical distribution function of the sample and the cumulative distribution function of the reference distribution. The null distribution of this statistic is calculated under the null hypothesis that the sample is drawn from the reference distribution.

The results of the goodness of fit tests could be summarized with their *Z* score; the lower its value, the closer the residuals to the expected pdf of the noise. Since the AIC and BIC criteria were indicators to be minimized, the *Z* scores of the goodness of fit tests could be included in their mathematical expressions as follows:

(7) $$AIC_{GF}=n\cdot \ln\left(\frac{MSE}{H}\left(1+Z_{score}^{2}\right)\right)+2k$$

(8) $$BIC_{GF}=n\cdot \ln\left(\frac{\sigma_{\varepsilon }^{2}}{H}\left(1+Z_{score}^{2}\right)\right)+kln\left(n\right)$$

The interpretation of this new version of the AIC and BIC criteria was very intuitive. The better the model, the lower the *Z* score of the residuals (since they are closer to the pdf of the noise) and, therefore, the lower the numerical value of the criteria. Equations (7) and (8) suggested also a consideration about the asymptote stability of AIC_GF_ and BIC_GF_ (where GF means Goodness of Fit); if the model was perfect, the *Z* score would tend to zero with increasing the number of points, and the AIC_GF_ and BIC_GF_ would converge to AIC_H_ and BIC_H_. The asymptotic converge of the criteria using the entropy of the residuals has already been demonstrated in [9], proving the asymptotic convergence of AIC_GF_ and BIC_GF_ as well. The AIC_GF_ and BIC_GF_ of Equations (7) and (8) were, therefore, the new versions of the model selection criteria that had been tested and whose performance is described in the rest of the paper.

## 3. Families of Functions and Noise Statistics

To investigate and quantify the performance of the alternative formulations of the model selection criteria, AIC_GF_ and BIC_GF_, a series of systematic tests were performed. The analysis was focused mainly on three classes of models, which were among the most useful and used in practice. They were the class of exponential functions, polynomials, and power laws. For each class, a representative example is discussed in the following. All the analyses were performed on bidimensional functions (*z* = *f*(*x*, *y*)).

The exponential functions investigated in this paper had the form:

(9) $$z\left(x,y\right)=axe^{\left(bx+cy\right)}+dxe^{\left(ex+fy\right)}+g$$

Polynomials are mathematical expressions that contain two or more algebraic terms, which can be added, subtracted, or multiplied. In general, polynomial expressions include at least one variable and typically also constants and positive exponents. Polynomial functions had the following form:

(10) $$z\left(x,y\right)=p_{00}+p_{10}x+p_{20}x^{2}+p_{01}y+p_{02}y^{2}+p_{03}y^{3}+p_{11}x+p_{21}x^{2}y+p_{12}xy^{2}$$

The power laws considered in this paper were monomials of the form:

(11) $$z\left(x,y\right)=cx^{a}y^{b}$$

where the exponents could be either positive or negative.

**Figure 1:**
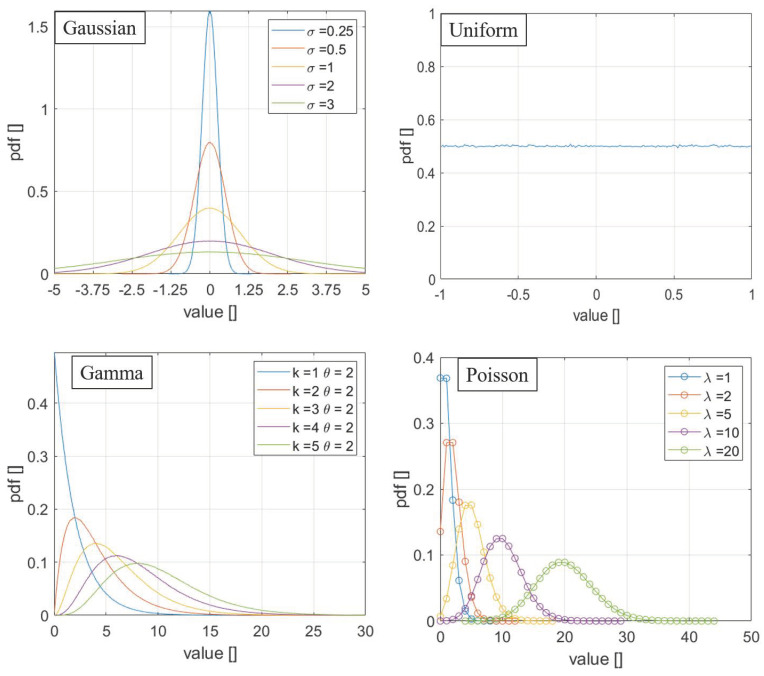
The probability density function of the four distributions used in the analyses reported in this paper.

With regard to the noise statistics, four of the most relevant distribution functions have been tested: Gaussian, uniform, Poisson, and gamma-distributed noise [14]. For the reader convenience, the mathematical formulation of these types of noise is reported in the following, while one representative case for each distribution is shown graphically in Figure 1.

Gaussian distribution:

$$f\left(x\right)=\frac{1}{\sigma \sqrt{2\pi }}e^{-\frac{1}{2}{\left(\frac{x-\mu }{\sigma }\right)}^{2}}$$

Uniform distribution:

$$f\left(x\right)=\frac{1}{b-a}\;for\;a\leq x\leq b$$

f(x)=0 otherwise

Poisson distribution:

$$f\left(x\right)=\frac{\lambda^{n}}{n!}e^{-\lambda }\;\forall \;n\in \;\mathbb{N}$$

Gamma distribution:

$$f\left(x\right)=\frac{x^{k-1}e^{-x/\theta }}{\theta^{k}\Gamma \left(k\right)}\;\forall x>0\;\mathrm{and}\;k,\theta \in \;\mathbb{N}$$

The parameters chosen for the examples reported in the paper were:

Gaussian distribution: *μ* = 0 *e σ* = 3.

Uniform distribution: *a* = −1 *e b* = 1;

Poisson distribution: *λ* = 10;

Gamma distribution: *k* = 3 *e θ* = 2.

## 4. Results for Exponential Functions, Polynomials, and Power Laws

All the analyses were performed by means of numerical tests. A systematic analysis of many cases for each class of functions had been performed. Given a domain of *x* and *y*, the function *z* = *f*(*x*, *y*) was built. Then, the noise with a specific distribution was added to the function. These data represented the points to fit.

To summarize the results in a concise way, in the following, for each class of functions, a representative case was reported. The values of the traditional AIC and BIC (Equations (3) and (4)) and of AIC_H_ and BIC_H_ were compared with those of AIC_GF_ and BIC_GF_ for two cases: the actual correct model, called “Correct”, and a very competitive alternative equation, called “Alternative”. The alternative equation had been obtained by fitting a different function to the data in such a way that the traditional indicator of the quality of the fit—RMSE and variance—were very competitive with the ones of the correct model. This fit had been performed with a nonlinear least-squares minimization method, which finds the parameters of a nonlinear equation by an iterative approach [15]. Being this method based on the minimization of the least squares, the function found was the one, which minimized also the classic versions of BIC and AIC. Therefore, the quality of the alternative criteria could be analyzed to see whether they showed better performance in the convergence to the right model, the one actually used to generate the synthetic data. The domain of each example was 0 < *x* < 10 and −10 < *y* < 10. A scan in the number of entries had been performed for all four types of noise distributions.

**Figure 2:**
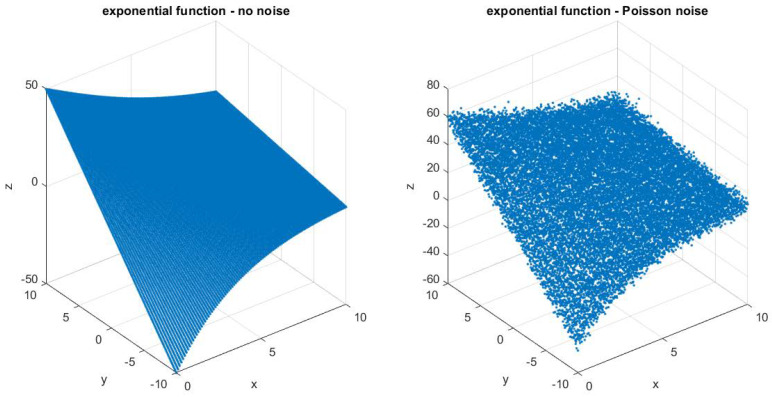
Exponential function scatter plot in the case of the no noise (**left**) and Poisson noise (**right**).

The exponential function used to generate the data was:

(12) $$z\left(x,y\right)=100\left(x+y\right)e^{-x}$$

while the function for the fit was the one given in Equation (9). A plot of the original function and with added noise of Poisson distribution is shown in Figure 2.

**Figure 3:**
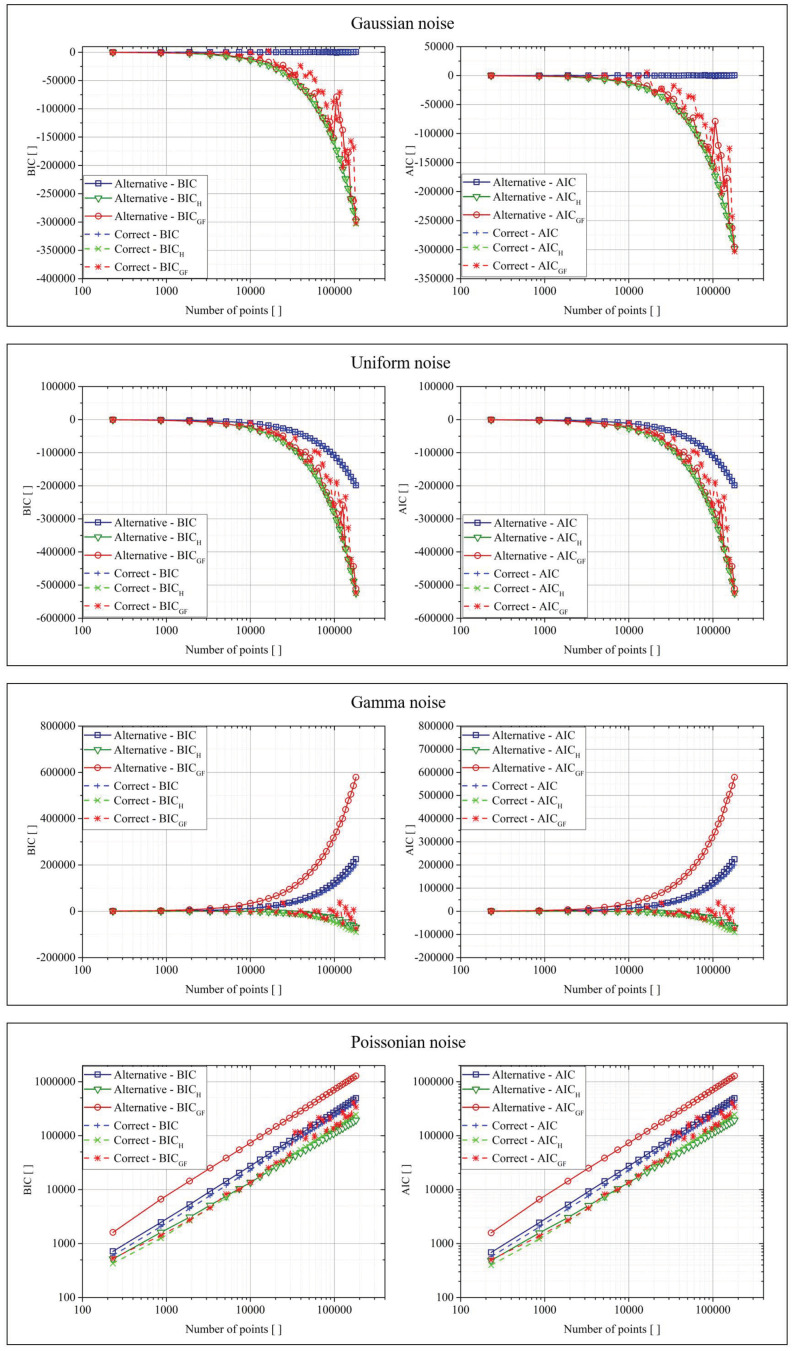
BIC (Bayesian information criterion) and AIC (Akaike information criterion) for the two fit functions in the case of the four different types of noise for the exponential function.

**Figure 4:**
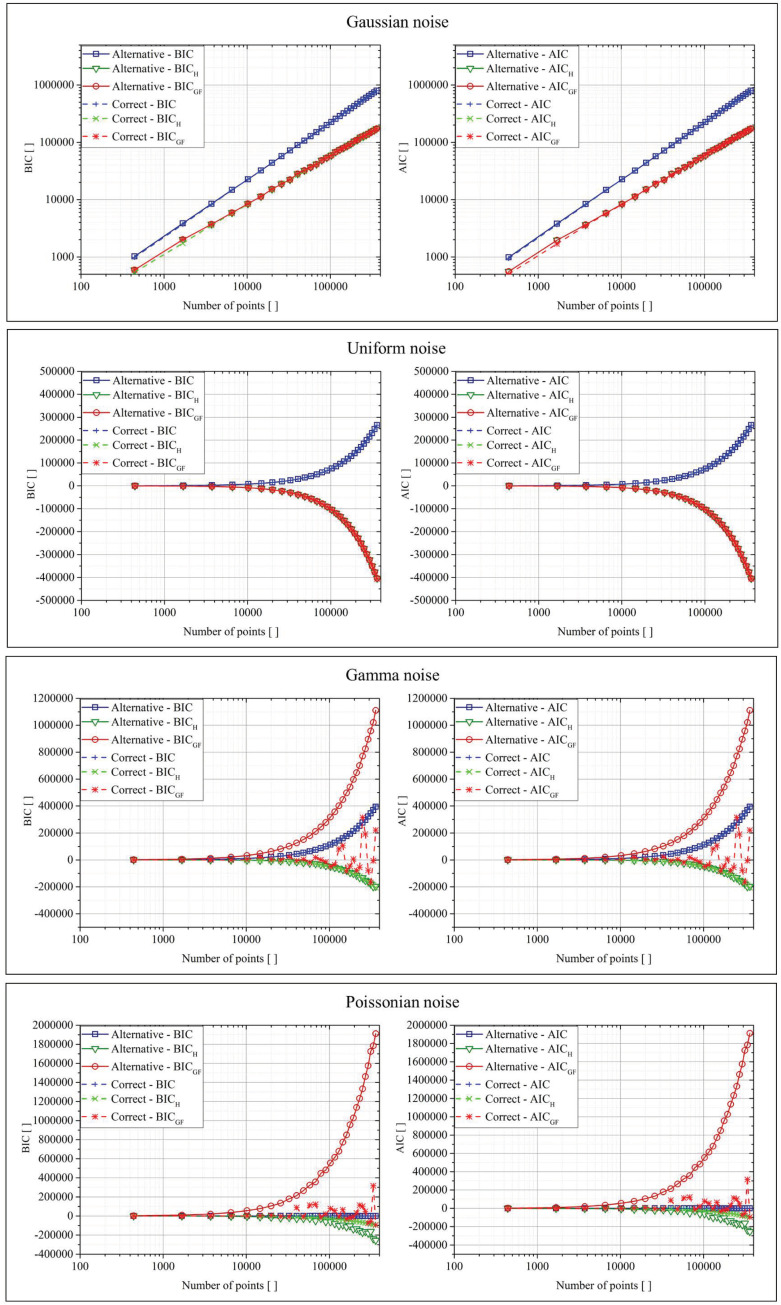
BIC and AIC for the two fit functions in the case of the four different types of noise for the polynomial function.

**Figure 5:**
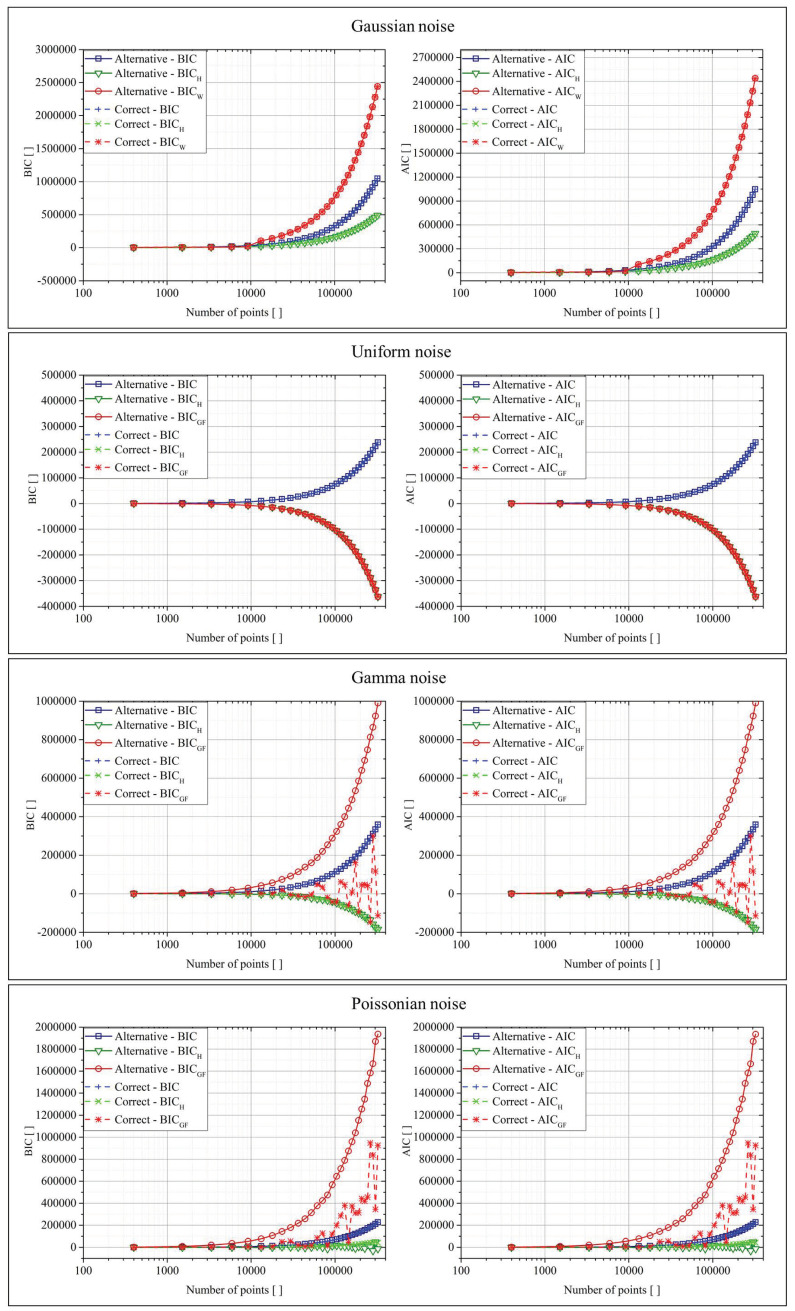
BIC and AIC for the two fit functions in the case of the four different types of noise for the power-law function.

**Figure 6:**
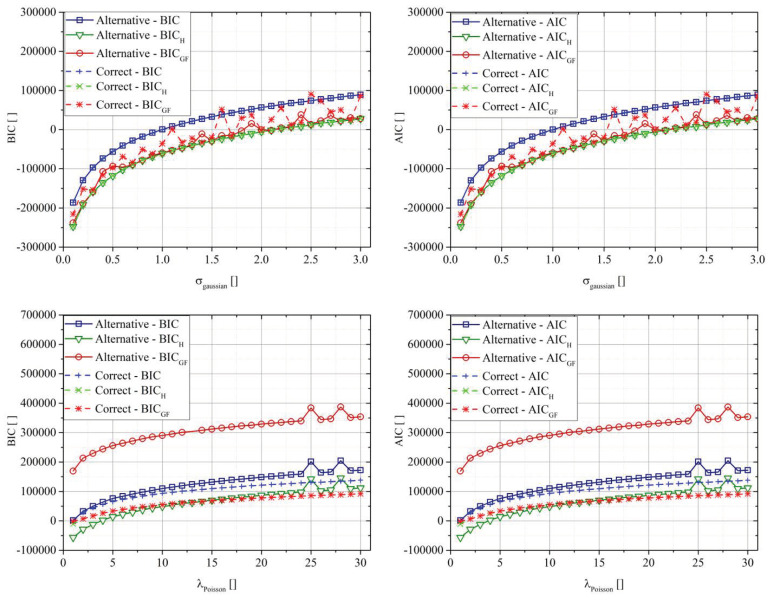
BIC and AIC for the two fit functions in the case of the four different types of noise for the power-law function.

Figure 3 reports the results for the exponential functions. In the plots of this figure, as in those of Figure 4, Figure 5 and Figure 6, the x-axis reported the number of points generated from the original function and used to test the various criteria. Moreover, each result was an average of over 10 repetitions of the calculations. The smoothness of the obtained curves was, of course, an implicit demonstration of their stability. Again on the nomenclature, the ”Correct” function referred to the original function used to generate the data (Equation (12)), while “Alternative” represented the function calculated by a regression tool, which was based on the minimization of the classic BIC and AIC. The tool used for the regression was the Curve Fitting Toolbox of Matlab, which allows performing both linear and non-linear fitting by using the least-square minimization method. For non-linear least squares, the robustness of the model could be achieved by the LAR (Least Absolute Residuals) or Bisquare approach, while the algorithms implemented were the Trust-Region and the Lavemberg–Marquardt.

The same analysis had been performed for polynomial type functions. The function used to generate the data was:

(13) $$z\left(x,y\right)=3x+x^{2}+y+3y^{4}$$

while the fit function was reported in Equation (10). The results are reported graphically in Figure 4.

The same analysis had been performed for power-law type functions. The function used to generate the data was:

(14) $$z\left(x,y\right)=cx^{3}y^{-1}$$

While the fit function was reported in Equation (11). The results are reported graphically in Figure 5.

The interpretation of the previously reported results was the same for all the classes of functions. For Gaussian and flat distributed noise, all versions of both AIC and the BIC (traditional formulation, Shannon entropy, and goodness of fit) returned basically equal values in all cases. As shown in [9], for these cases, the upgraded versions—AIC_H_, BIC_H_ and AIC_GF_, BIC_GF_—improve only the convergence to the right model in limited and very difficult cases. For the other two types of noise, the situation was dramatically different. The use of the “goodness of fit” approach gave a much smaller BIC_GF_ and AIC_GF_ in the case of the correct function, improving systematically the convergence to the right model. Basically, this result was due to the fact that, in the traditional AIC and BIC, the minimization of the errors automatically implies that the fit acts with the purpose of minimizing the mean of the errors (by bringing it as close as possible to zero). This requirement was not appropriate in the case of data affected by the noise of Poisson and gamma distributions, which were non-symmetric with respect to zero. For these cases, the goodness of fit tests significantly improved the capability of the criteria to select the right model. In general, BIC_GF_ and AIC_GF_ are expected to provide appreciable better performance in all cases whose data are affected by no zero-sum distributed noise. Concerning the rate of convergence and the effects of data size, the most important thing to consider is that all goodness of fit tests need an adequate amount of data in order to return a reliable Z-score. On the other hand, from the plots in the previous figures, it could be seen how AIC_GF_ and BIC_GF_ provided better performance than AIC and BIC, even for a quite limited number of points, of the order of 100, which is a very real demand in most practical applications. Therefore, the rate of convergence of the proposed criteria seemed also to be quite satisfactory.

## 5. Effect of Noise Intensity

Figure 6 compares the various BIC and AIC definitions in the case of the exponential function, changing the intensity of two types of noise, Gaussian and Poisson. In the case of the Gaussian distribution, the parameter was the standard deviation of the distribution, while in the case of the Poisson, the parameter λ.

The parametric analysis validated the previous results for any tested noise intensity. In the case of Gaussian distributions, the use of the GF approach did not lead to real improvements. In the case of the Poisson distribution, both AIC_GF_ and BIC_GF_ significantly improved the selection criteria, leading to a large difference between the “Correct” and the “Alternative” cases, independently from the noise intensity.

## 6. Conclusions

The most widely used versions of the model selection criteria AIC and BIC are valid under the assumption that the data are affected by Gaussian, zero-sum additive noise. This is a consequence of the fact that, in most practical applications in science and engineering, often it is very difficult, if not impossible, to compute the likelihood of the data given the model. In this situation, the AIC and BIC have been reformulated as reported in Equations (3) and (4), in which the statistical information about the residuals is completely summarized by their MSE and variance. If the data is affected by noise non-symmetric with respect to zero, these versions of the criteria can fail badly because they favor models with zero-sum residuals. On the contrary, the new proposed versions of the selection criteria, AIC_GF_ and BIC_GF_, present significantly better performance because they take into account the fact that the residuals should present a probability distribution function as close as possible to the one of the noise. Indeed, all the numerical tests performed indicate that, if the noise distribution is known and sufficient amount of data are availed to compute reliably the *Z* scores, AIC_GF_ and BIC_GF_ are the versions of the criteria to rely on. Under these conditions, they have always proved to have much better convergence properties in all respects. As a consequence, the proposed new versions of the selection criteria are expected to be deployed quite systematically in various fields of complex science, ranging from high-temperature plasmas [16,17,18,19,20,21,22,23] to atmospheric physics [24,25]. Another interesting application could be found in the regularization of recent tomographic inversion methods [26,27,28].

From a methodological standpoint, it should be mentioned that the upgraded versions, AIC_GF_ and BIC_GF_, include frequentist indicators in information-theoretic and Bayesian criteria. This approach seems quite promising and worth further investigations. The proposed criteria could also be further improved by implementing more advanced metrics, such as the geometric distance and the Venn definition of probability [29,30,31]. In this perspective, a combination of the proposed upgrades with recently developed extensions of the AIC criterion, using a class of pseudo distances instead of the Kullback-Leiber divergence, should also be seriously pursued [32].
